# Therapeutic consequence of adrenal venous sampling complication: remission of primary aldosteronism after adrenal vein thrombosis

**DOI:** 10.1210/jcemcr/luag243

**Published:** 2026-09-10

**Authors:** Navjot Kaur Grewal, Colin Carracher, Andrew Welch, Yiyang Lu

**Affiliations:** Division of Endocrinology, Diabetes and Metabolism, University of Cincinnati, College of Medicine, Cincinnati, OH 45202, USA; Division of Endocrinology, Diabetes and Metabolism, University of Cincinnati, College of Medicine, Cincinnati, OH 45202, USA; Division of Endocrinology, Diabetes and Metabolism, University of Cincinnati, College of Medicine, Cincinnati, OH 45202, USA; Department of Pathology, University of Cincinnati, College of Medicine, Cincinnati, OH 45202, USA

**Keywords:** primary aldosteronism, adrenal vein thrombosis, adrenal venous sampling

## Abstract

A 43-year-old man with resistant hypertension and hypokalemia was diagnosed with primary aldosteronism (PA), confirmed by biochemical testing and adrenal vein sampling (AVS), which lateralized aldosterone secretion to the right adrenal gland. Shortly after AVS, he developed right flank pain, and imaging showed mild postprocedural changes without overt complications. Three months later, he presented with hyperkalemia and acute kidney injury, but notably, his blood pressure had improved with fewer antihypertensives. Laboratory evaluation revealed suppressed aldosterone, normal renin, and no evidence of catecholamine excess. Imaging and hormonal reassessment suggested spontaneous resolution of PA. Despite plans for adrenalectomy based on initial AVS results, surgery revealed a thrombosed right adrenal vein and infarcted adrenal tissue. Histopathology confirmed hemorrhagic infarction with chronic inflammation. This rare case highlights adrenal vein thrombosis during AVS, inducing an acute, transient biochemical remission of PA via infarction of the aldosterone-producing gland. It underscores the importance of vigilant post AVS monitoring and recognition that procedural complications, while uncommon, may yield unexpected therapeutic benefit in select patients.

## Introduction

Primary aldosteronism (PA) remains significantly underdiagnosed, despite its known contribution to hypertension and end-organ damage. Timely identification and management of PA are essential, given its strong association with increased cardiovascular and renal morbidity and the potential for surgical cure [1]. Adrenal vein sampling (AVS) is the gold standard diagnostic procedure for distinguishing between unilateral and bilateral sources of aldosterone excess [2]. While generally safe, AVS carries a small risk of serious complications. We present a unique case in which an AVS-related complication of adrenal vein thrombosis led to infarction of the aldosterone-producing adrenal gland, inducing an acute, temporary remission of the patient's PA. This case adds novel insights to the literature by documenting a rare and complex procedural complication.

## Case presentation

A 43-year-old man was referred for evaluation of resistant hypertension and recurrent hypokalemia. His medical history was notable for obstructive sleep apnea, obesity, functional hypogonadotropic hypogonadism, and chronic hypertension. He reported symptoms of occasional headaches, fatigue, low libido, and weight gain, attributed to a sedentary lifestyle and suboptimal dietary habits. He denied any history of skin changes, cardiovascular events, tremors, palpitations, or diaphoresis.

His hypertension had been managed with amlodipine, benazepril, atenolol, and chlorthalidone for nearly a decade, along with intermittent potassium supplementation.

## Diagnostic assessment

On examination, he appeared well, with findings limited to morbid obesity. There were no features suggestive of Cushing syndrome. Thyroid examination was unremarkable, and peripheral pulses were normal. Testicular exam showed normal volume with no abnormalities.

**Figure 1:**
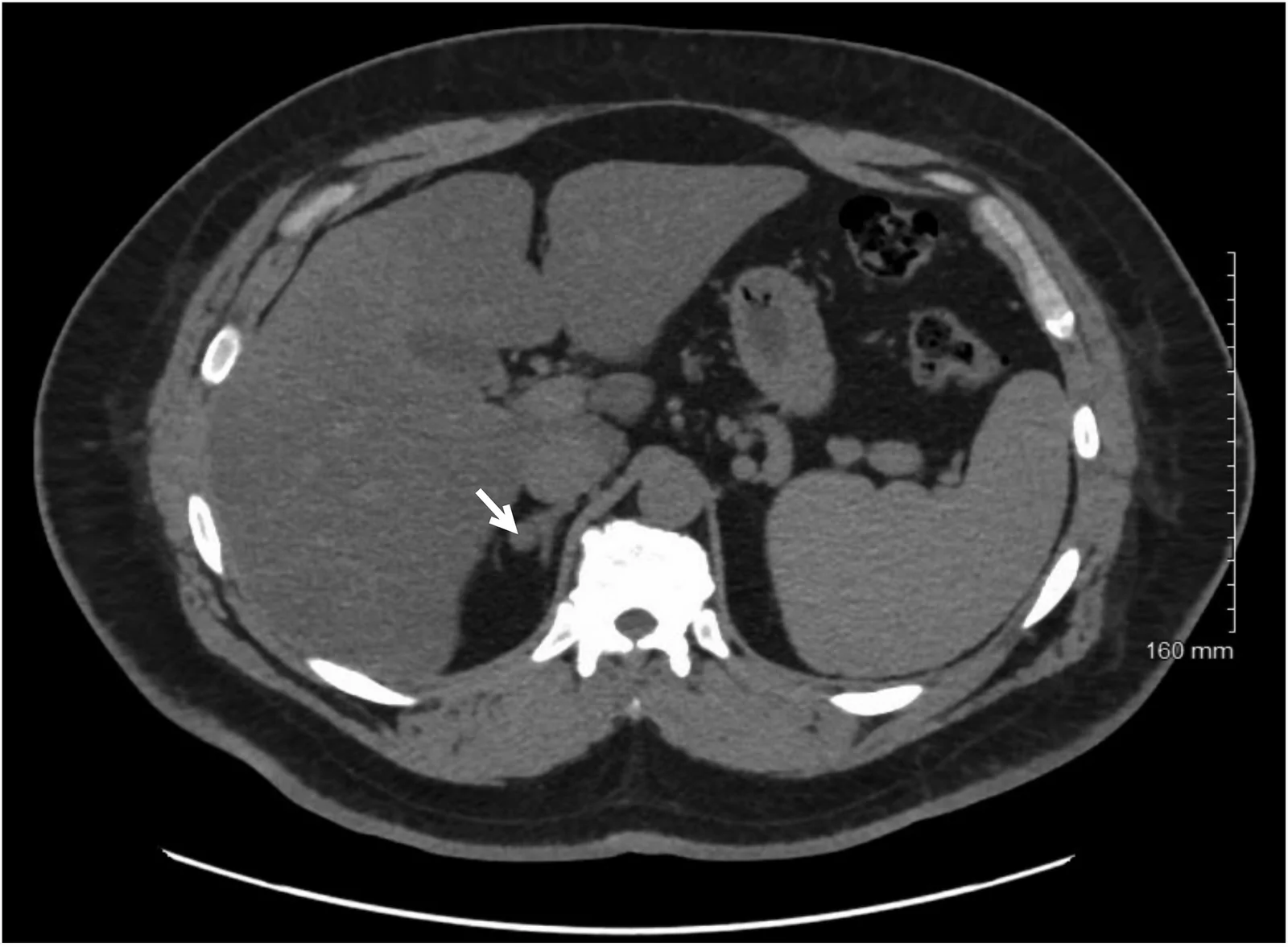
Right adrenal nodule with an unenhanced computed tomography attenuation of 18.4 Hounsfield units.

Initial laboratory evaluation revealed elevated plasma aldosterone levels with suppressed renin activity (Table 1). A saline suppression test confirmed the diagnosis of PA (aldosterone, 9.9 ng/dL [274 pmol/L]; reference range, <3.0-23.2 ng/dL [<83-643 pmol/L]), close to the diagnostic threshold of >10 ng/dL (277.4 pmol/L) [3]. Computed tomography (CT) identified a 1.2 cm right adrenal nodule with an unenhanced CT attenuation of 18.4 Hounsfield units (HU); the left adrenal gland appeared normal (Fig. 1).

**Table 1:** Trend of renin/renin activity and aldosterone

	Reference ranges	November 2023 (pre-AVS)	December 2024 (post-AVS)	February 2025 (preadrenalectomy)
Renin, ng/mL/h	0.167-5.380 ng/mL/h	0.557 ng/mL/h	0.877 ng/mL/h	3.472 ng/mL/h
Aldosterone, ng/dL (pmol/L)	<3.0-23.2 ng/dL (<83-643 pmol/L)	13.0 ng/dL (360 pmol/L)	<3 ng/dL (<83 pmol/L)	9.8 ng/dL (271.9 pmol/L)
Potassium, mmol/L	3.5-5.3 mmol/L	3.5 mmol/L	6.0 mmol/L	4.8 mmol/L

The patient remained on a beta-adrenergic blocker during aldosterone and renin ratio assessment, which can suppress renin and increase the likelihood of false-positive results. This effect is most relevant when aldosterone concentrations are in the intermediate range (10-15 ng/dL [277-416 pmol/L] by immunoassay or 7.5-10 ng/dL [208-277 pmol/L] by LC-MS). However, when aldosterone levels exceed these thresholds, as in this case, the diagnosis of primary aldosteronism remains likely despite concurrent beta-adrenergic blockade [3]. The patient was initiated on a mineralocorticoid receptor antagonist and advised taking potassium supplements while awaiting AVS. The AVS procedure confirmed right-sided lateralization of aldosterone secretion (Table 2).

**Table 2:** Adrenal vein sampling with cosyntropin results

Vein	Aldosterone, ng/dL	Cortisol, μg/dL	Selectivity index, SI^a^	Aldosterone/cortisol ratio^b^	Lateralization index^c^	Contralateral suppression index^d^
Sample 1						
RT adrenal	3450 ng/dL	1670 μg/dL	69.58	2.07	39.05	
LT adrenal	41 ng/dL	775 μg/dL	32.29	0.05		0.07
Inf vena cava	18 ng/dL	24 μg/dL		0.75		
Sample 2						
RT adrenal	13300 ng/dL	1390 μg/dL	55.60	9.57	170.97	
LT adrenal	38 ng/dL	679 μg/dL	27.16	0.06		0.09
Inf vena cava	16 ng/dL	25 μg/dL		0.64		

^a^Adrenal vein cortisol-to-IVC cortisol ratio; a value >5 indicates successful cannulation.

^b^Used to normalize aldosterone to cortisol secretion.

^c^High-side aldosterone/cortisol to low0side aldosterone/cortisol ratio; a value >4 indicates unilateral aldosterone production.

^d^Low-side aldosterone/cortisol to IVC aldosterone/cortisol; a value <1 indicates contralateral aldosterone suppression.

## Treatment

**Figure 2:**
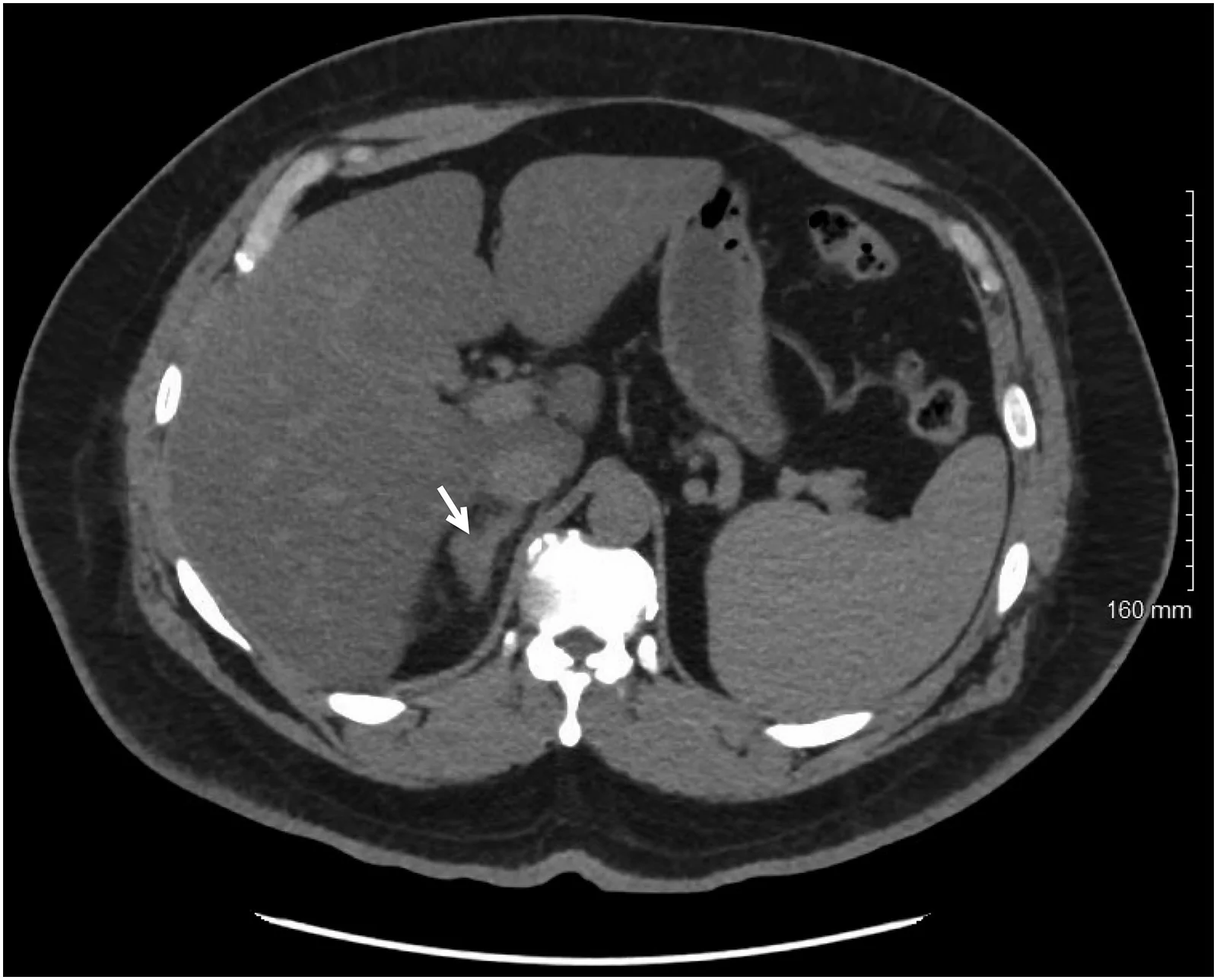
Computed tomography abdomen pelvis without contrast: mild fat stranding surrounding the right adrenal gland, most likely postprocedural. No evidence of significant hematoma or active contrast extravasation.

Shortly after AVS, the patient experienced right flank pain and was admitted for observation. CT imaging revealed mild fat stranding near the right adrenal gland, interpreted as post AVS changes without evidence of hematoma or contrast extravasation (Fig. 2). He was discharged with low-dose oxycodone, potassium supplements, and a regimen including amlodipine, atenolol, lisinopril, and hydrochlorothiazide. He subsequently missed his follow-up endocrinology visit.

**Figure 3:**
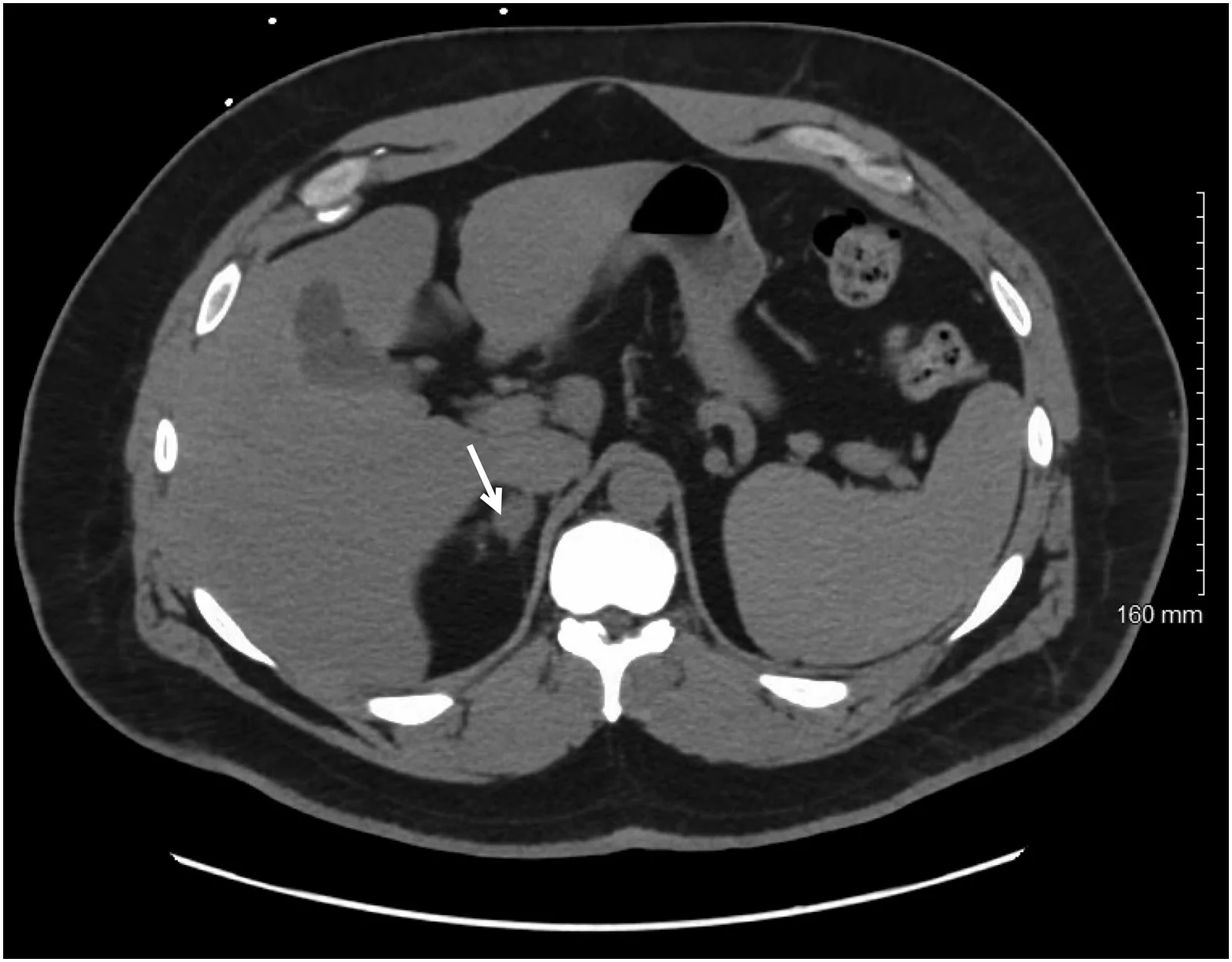
Computed tomography abdomen pelvis without contrast: stable right adrenal nodule with mild periadrenal stranding, obtained 3 months following adrenal vein sampling procedure.

Three months later, the patient presented with myalgias and fatigue. Laboratory testing showed hyperkalemia and acute kidney injury (prerenal), attributed to dehydration, use of potassium supplements, and nonsteroidal anti-inflammatory drug intake. During this hospitalization, he displayed a notable improvement in blood pressure control on a reduced antihypertensive regimen. Repeat laboratory tests demonstrated suppressed aldosterone, low renin activity (Table 1), and adequate morning cortisol (13.1 μg/d [361 nmol/L], reference range, 6.7-22.6 μg/dL [185-624 nmol/L]). Plasma fractionated metanephrines were also normal. Follow-up imaging showed only mild post AVS changes (Fig. 3). The patient was discharged on amlodipine and atenolol. Lisinopril and hydrochlorothiazide were discontinued due to kidney injury. Repeat testing prior to his previously planned adrenalectomy revealed normalized aldosterone secretion with renin-aldosterone physiology intact, suggesting contralateral adrenal recovery (Table 1). The temporal association between the AVS procedure and subsequent clinical course suggested that adrenal vein thrombosis or injury occurred during AVS, leading to infarction of the aldosterone-secreting adrenal nodule. The resulting cessation of aldosterone hypersecretion explained the normalization of hormonal markers and improved clinical status.

## Outcome and follow-up

**Figure 4:**
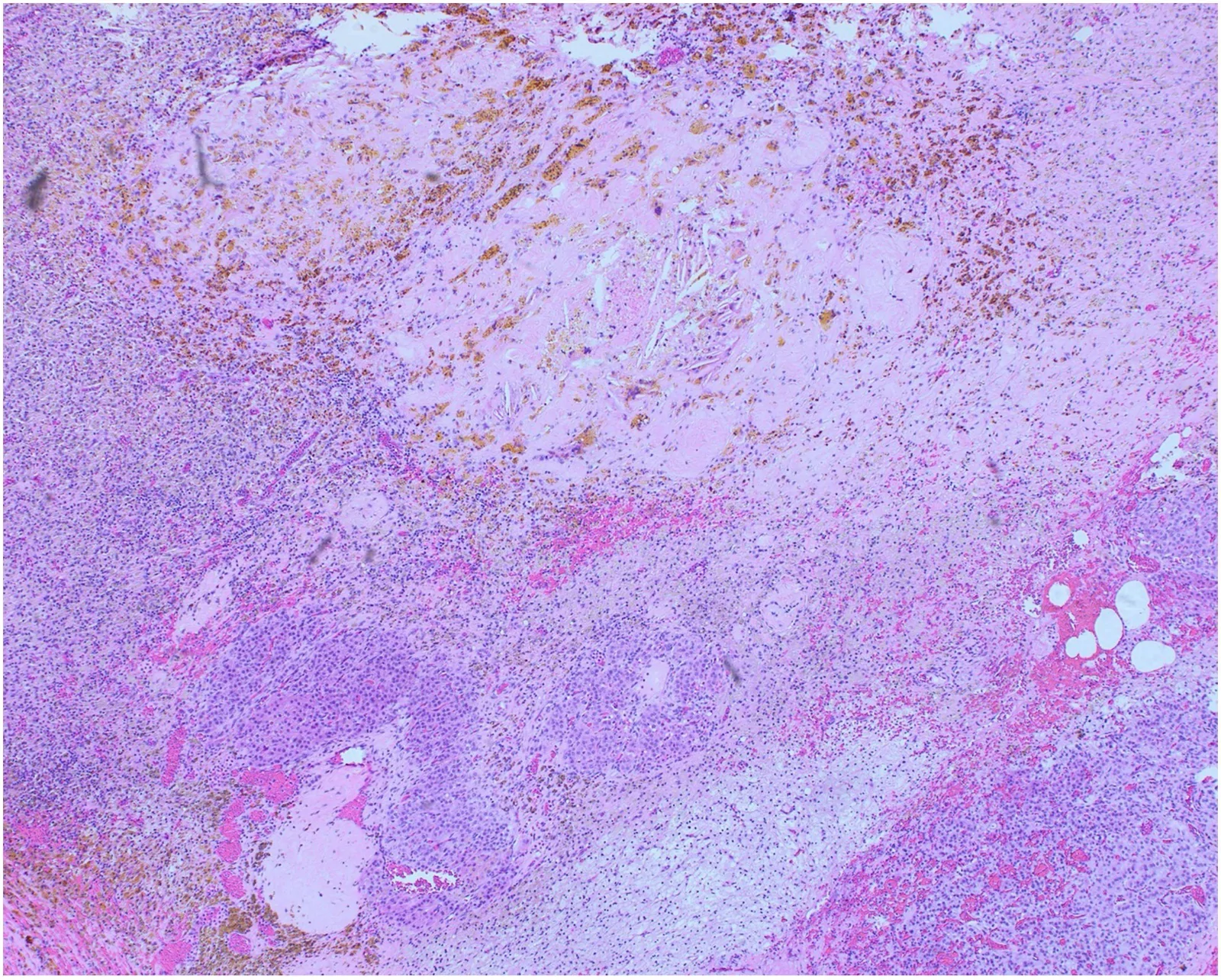
Magnification 4×. Hematoxylin and eosin section shows granulomatous inflammation composed of foamy histiocytes, cholesterol clefts, hemosiderin microphages, consistent with hemorrhagic infarction. There are residual normal adrenal cortical cells at the lower periphery of the infarction foci.

Despite biochemical remission, conservative management vs surgery was discussed in detail. The patient was informed that adrenalectomy was not medically required given apparent resolution of PA. However, due to the unpredictable nature of the long-term durability of a postthrombotic remission and risk of delayed clinical recurrence, and to ensure definitive, long-term hormonal control, unilateral adrenalectomy was pursued via shared clinical decision-making. This clinical approach is strongly supported by historical cohorts demonstrating that hyperfunctioning adenomatous tissue routinely survives localized adrenal injuries [4]. Intraoperatively, the gland was observed to be infarcted and surrounded by inflammatory adhesions. The adrenal vein was thrombosed with adjacent scar tissue. Pathology confirmed hemorrhagic infarction, granulomatous inflammation, foreign body giant cell reaction, and cholesterol clefts, supporting adrenal vein thrombosis with resultant infarction as the etiology of resolution (Fig. 4). No malignancy was identified.

**Figure 5:**
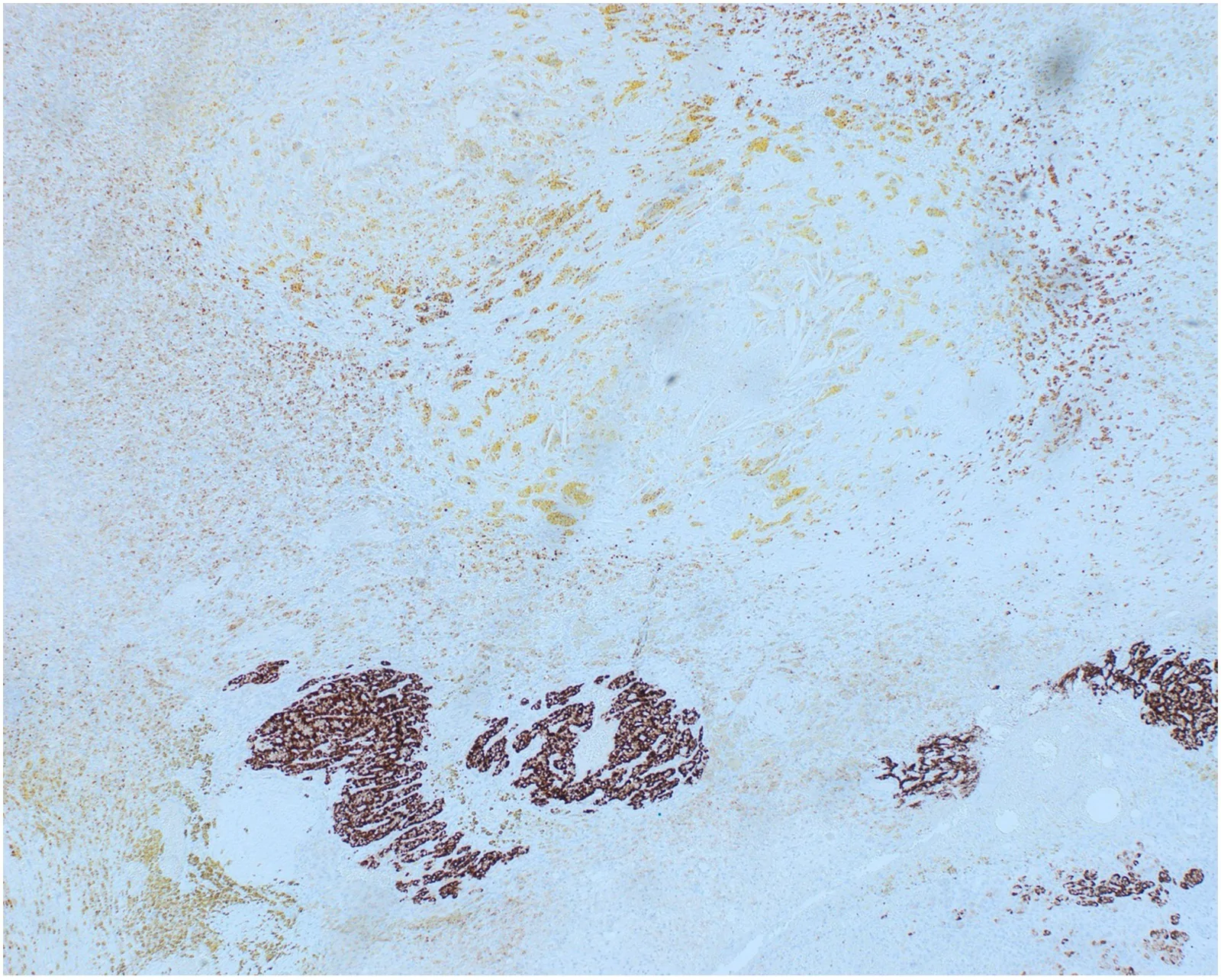
Magnification 4×: CYP11B2 IHC stain: there are residual normal adrenal cortical cells at the lower periphery of the infarction foci, which are highlighted by CYP11B2 immunohistochemistry demonstrating aldosterone synthase expression.

To further investigate the functional status of the gland and address whether the AVS-induced infarction had achieved complete biochemical resolution, immunohistochemistry (IHC) for CYP11B2 (aldosterone synthase) was performed. The IHC analysis revealed that while the majority of the adrenal architecture had undergone hemorrhagic infarction, there were distinct clusters of residual normal adrenocortical cells scattered in between the infarction foci. Crucially, these residual cells demonstrated strong expression of CYP11B2, confirming the persistence of aldosterone-producing tissue despite the extensive procedural injury (Figs. 4 and  5). The postoperative identification of these aldosterone synthase-producing cells retrospectively validated the clinical decision to operate. It provided histopathological proof that the AVS complication resulted in an incomplete auto-adrenalectomy, confirming the preoperative suspicion that autonomous cells remained in situ and could have eventually driven a clinical relapse.

Postoperatively, the patient remained normokalemic with controlled blood pressure on a reduced regimen of amlodipine and atenolol, consistent with sustained resolution of aldosterone excess.

## Discussion

The majority of hypertensive patients are presumed to have essential hypertension, often resulting in underdiagnosis of secondary causes such as PA. However, when properly screened, PA is more common than previously believed and remains a potentially curable cause of hypertension that reduces morbidity when treated [2].

AVS is the cornerstone for identifying lateralized aldosterone production. Though rare, complications such as adrenal vein rupture, hemorrhage, and thrombosis may occur and are usually associated with morbidity. In rare cases, however, such complications may induce an acute biochemical remission [5, 6].

Our case documents spontaneous resolution of PA following adrenal infarction caused by AVS-induced adrenal vein thrombosis. Because a definitive unilateral adrenalectomy was pursued to secure long-term hormonal control, the natural timeline of this complication was intentionally interrupted. However, historical data heavily imply that such remissions face a high risk of being temporary. Monticone et al [4] examined a retrospective cohort of 24 cases of adrenal hemorrhage during AVS and demonstrated that even when structural damage occurs in the gland ipsilateral to an aldosterone-producing adenoma, long-term reduction in blood pressure or permanent biochemical resolution of primary hyperaldosteronism is typically not achieved, confirming that hyperfunctioning adenomatous tissue routinely survives localized vascular insults. In one report by Okamura et al [7], a patient developed adrenal hematoma following AVS but did not experience improvement in aldosterone levels or blood pressure. In contrast, Zulfiqar et al [8], described a case where AVS-related hemorrhage rendered an adrenal lesion nonfunctional, resulting in resolution of PA and withdrawal of antihypertensive medications. While our case uniquely demonstrated clinical and biochemical response in the acute post-procedural phase, proceeding with definitive surgery addresses the latent risk of adenoma recovery highlighted by these historical cohorts, rather than waiting for an unpredictable clinical relapse to occur.

The mechanism of adrenal vein thrombosis in this case is likely multifactorial. The right adrenal vein is anatomically small and technically challenging to cannulate, rendering it susceptible to endothelial injury during catheter manipulation and contrast injection. Postprocedural flank pain and imaging findings of periadrenal fat stranding support localized vascular injury. Mechanical trauma, contrast-induced endothelial damage, or transient venous stasis may have contributed to thrombosis and subsequent infarction of aldosterone-producing tissue [5]. Of note, hematological and prothrombotic factors were not evaluated during the acute admission, which represents a limitation in fully characterizing the patient’s underlying baseline hypercoagulability risk.

CYP11B2 (aldosterone synthase) IHC is a highly specific marker for identifying functionally autonomous aldosterone-producing cells, which can be missed on standard hematoxylin and eosin staining [9]. The resected gland demonstrated that despite extensive hemorrhagic infarction, viable clusters of CYP11B2-expressing cells persisted at the periphery of the necrotic foci.

This histopathological finding provides critical insight for managing similar AVS complications in the future. The presence of residual aldosterone synthase-producing cells demonstrates that spontaneous remission via infarction can be incomplete, masking a persistent risk for delayed recurrence of hyperaldosteronism [10, 11].

The patient with acute kidney injury is most consistent with a prerenal etiology in the setting of volume depletion from dehydration and concurrent nonsteroidal anti-inflammatory drug use. However, resolution of hyperaldosteronism may also have contributed. Chronic aldosterone excess promotes intravascular volume expansion and glomerular hyperfiltration; abrupt correction can lead to relative volume contraction and a decline in glomerular filtration rate [10].

AVS findings further support the preinfarction presence of unilateral disease. A contralateral suppression index <1 indicated suppression of the left adrenal gland, while subsequent normalization of renin and aldosterone levels reflects recovery of contralateral adrenal function following infarction. This highlights the dynamic nature of the renin-angiotensin-aldosterone system after abrupt correction of aldosterone excess [10].

Recent advances have introduced minimally invasive alternatives to adrenalectomy, including radiofrequency ablation for aldosterone-producing adenomas. These approaches aim to selectively ablate hyperfunctioning tissue while preserving adrenal function. In contrast, this case represents an unintentional but analogous outcome, where AVS-induced vascular injury resulted in functional ablation of aldosterone-producing tissue [12]. However, unlike controlled thermal ablation, AVS-induced infarction is unpredictable in its distribution. As evidenced by our IHC results, peripheral cortical tissue may remain well-perfused enough to harbor residual autonomous cells [9, 13].

Our case contributes novel documentation of AVS-induced infarction resulting in acute biochemical and clinical remission of PA. By utilizing CYP11B2 immunohistochemistry, we have demonstrated that clinical remission post-complication does not equate to complete pathological resolution. The findings stress the importance of close post-procedural follow-up and a proactive approach.

## Learning points

- Primary aldosteronism is frequently underdiagnosed and is a potentially curable cause of secondary hypertension when identified early.
- Adrenal vein sampling remains the gold standard for lateralizing aldosterone secretion and guiding management.
- Though rare, complications from AVS such as adrenal vein thrombosis can lead to infarction of the affected gland.
- Such complications, while often associated with morbidity, may paradoxically resolve aldosterone excess, providing a curative outcome.
- Recognition and reporting of these rare outcomes can enhance clinical awareness and inform future patient management.

## Contributors

All authors made individual contributions to authorship. N.K.G., C.C., and A.W. were involved in the diagnosis and management of the patient; Y.L. was involved in the histopathology section and preparation of histology images; and N.K.G. was involved in manuscript submission. All authors reviewed and approved the final draft.

## Data Availability

Data sharing is not applicable to this article as no datasets were generated or analyzed during the current study.
